# MiR-34a affects dexmedetomidine-inhibited chronic inflammatory visceral pain by targeting to HDAC2

**DOI:** 10.1186/s12871-019-0801-z

**Published:** 2019-07-19

**Authors:** Meng Liang, Aijie Shao, Xinsheng Tang, Meiling Feng, Jing Wang, Yingna Qiu

**Affiliations:** Department of Anesthesiology, Weihai Central Hospital, No.3, West Mt. East Road, Wendeng District, Weihai City, 264400 Shandong China

**Keywords:** CIVP, DEX, miR-34a, HDAC, Pain behaviors

## Abstract

**Background:**

Dexmedetomidine (DEX) has been used as an anesthetic for decades. The present investigation aimed to elucidate the analgesic impact of DEX on 2,4,6-Trinitrobenzenesulfonic acid (TNBS)-induced chronic inflammatory visceral pain (CIVP) in rats.

**Methods:**

TNBS with or without DEX to Male Sprague-Dawley SD rats were randomly divided into four groups: normal, CIVP, DEX, and vehicle. Pain behaviors were assessed and the abdominal withdrawal reflex, mechanical withdrawal threshold, and thermal withdrawal latency were recorded. Quantitative polymerase chain reaction data showed increased expressions of pro-inflammatory cytokines (IL-6, IL-1β and TNF-α) in the spinal cord tissues of rats.

**Results:**

RNA microarray and quantitative polymerase chain reaction results indicated that miR-34a was downregulated by TNBS induction, but it was upregulated by DEX administration. Further studies showed that transfection of adenovirus-miR-34a inhibitor reversed the effect of DEX on the pain behaviors and spinal-cord pro-inflammatory-cytokine generation in CIVP rats. Additionally, we found that miR-34a targeted the 3′-UTR of the HDAC2 gene, as evinced by the increased HDAC2 expression in the CIVP and DEX + miR-34a inhibitor groups, and decreased HDAC2 signaling in the DEX group. Moreover, knock-down of HDAC2 restored DEX-attenuated pain behaviors and reduced pro-inflammatory cytokine production.

**Conclusions:**

DEX thus exhibited an analgesic effect on CIVP rats through the miR-34a-mediated HDAC2 pathway and suppressed visceral hypersensitivity.

## Background

Around 10–40% of the population experiences regular visceral pain [1]. Visceral pain is a major threat to an individual’s health. Uncertain localization and repeated occurrence of referred pain are features of inflammatory visceral pain. Visceral pain was previously thought to involve various neurophysiological mechanisms and transmission pathways [2]. Unfortunately, only a few analgesics specific to the management of visceral pain are available, among which opioids have an become indispensable treatment option. Nevertheless, constipation, urine retention, vomiting, respiratory depression, nausea, and other dose-dependent side effects are observed in patients receiving opioids.

Morris et al. [3] maintained that the intra-rectal administration of 2,4,6-trinitrobenzene sulfonic acid (TNBS) and 100 mg/kg of ethanol to rats could induce colitis. Ethanol was found to promote the TNBS-mediated compromising of intestinal tissues and colon-tissue proteins [4, 5]. TNBS-related colitis is characterized by a progression of transmural inflammation that is highly similar to the histopathological injury observed in Crohn’s disease, rendering TNBS-induced inflammation of the colon as a good model of Crohn’s disease [6].

Detomidine and xylazine are routinely applied by physicians and veterinarians [7]. Dexmedetomidine (DEX), also used clinically for sedation, binds to α2-receptors with an eight-fold higher affinity than does clonidine [8]. It has also been reported that the analgesic effects of DEX are stronger than those of clonidine, and the former’s antinociceptive reportedly also occurs following intraspinal application [9]. DEX exhibited an analgesic effect on TNBS-induced chronic inflammatory visceral pain (CIVP) in rats through a miR-211-mediated MEK/ERK/CREB pathway, suppressing visceral hypersensitivity [9]. DEX is reportedly able to some extent replace the usage of fentanyl in laparoscopic bariatric surgery [10] and abdominal hysterectomy [11]. However, there has hitherto been a paucity of data on the capability of DEX to alleviate chronic inflammatory visceral pain as well as on a mechanism underlying this capacity.

Micro ribonucleic acids (miRNAs) are RNAs comprised of 22–25 nucleotides that lack any coding ability but are able to regulate the expression of proteins at the post-transcription level [12]. Several studies have highlighted the crucial influence of miRNAs on multiple biological reactions: the expression of miR-34a is suppressed in malignancies and cellular processes such as cell differentiation, proliferation, and death [13–15], and miR-34a was shown to contribute to senescence regulation triggered by ionizing radiation in human non-small cell lung cancer cells [16]. Prior research has also found that miR-34a influences the invasion and migration of osteosarcoma cells and that miR-34a exhaustion could enhance the expression pattern of cells, resulting in osteosarcoma metastasis [17]. However, more efforts are needed to elucidate the mechanisms underlying miR-34a-mediated pain-related biological processes. Obvious pain in the viscera might occur after the intraperitoneal injection of TNBS; this method has been used to model visceral pain in animals by many studies [18]. The present study investigated miRNA profiling in the spinal cord under TNBS-induced chronic inflammatory visceral pain using a rat model and explored the influence of miR-34a-HDAC2 on the behaviors resultant of visceral pain in the spinal cord.

## Methods

### Animals

A total of sixty adult male Sprague Dawley (SD) rats (obtained commercially from Vitalriver, China; average weight, 400 g) were used. HDAC2^−/−^ rats were purchased from Nanjing Biomedical Research Institute of Nanjing University (NBRI), and the genetic background is Sprague Dawley. The rats were placed were housed under a 12/12 h light/dark cycle where Food and water was available ad libitum except before testing: they were deprived of food and water for 24 h prior to electrophysiological and behavioral experiments [19–21]. Rats were anesthetized with Nembutal (45 mg/kg i.p.), then continued exposure to CO2 for at least 15 min was utilized for rat euthanasia. All animal handling and experimental protocols were approved by an institutional animal care and use committee in Weihai Central Hospital.

### TNBS-induced CIVP

TNBS was applied to promote CIVP in the colon of rats as previously described [3]. The 60 SD rats were randomly divided into four groups of 15 rats each: normal (no treatment), CIPV (TNBS-treated rats), 0.3 ml of 400 μg/ml DEX (Sigma-Aldrich, DEX; 0.3 mg/kg DEX in saline with 5% DMSO), and 0.3 ml of vehicle (saline with 5% DMSO) group.

Briefly, 15 min after the subdural injection of saline or DEX, the rats were anesthetized with intraperitoneally administered pentobarbital sodium (dose, 50 mg/kg). Using a 16-Gauge gavage needle, 0.5 mL of 50% TNBS (diluted 1:1 in ethanol) was slowly injected into the descending colon. Through the biochemical detection of myeloperoxidase activity in the colon and the histopathological technique of marking colon tissues with hematoxylin and eosin staining, we confirmed the beginning of inflammation in the tested rats. Every procedure was approved by the Animal Care and Use Committee of Weihai Central Hospital.

### Abdominal withdrawal reflex (AWR), mechanical withdrawal threshold (MWT), and thermal withdrawal latency (TWL) measurements

As described previously [18], the AWR was determined using colorectal distending (CRD) with four grades of stimuli at 2 weeks after TNBS induction: 80, 60, 40, and 20 mmHg. The stimulation time of each CRD was 20 s, and there was five-minute interval between two stimuli. Three independent measurements were obtained and their average value was calculated.

Because TNBS treated rats demonstrated somatic hypersensitivity in response to somatic stimuli of the hind-paw [22]. MWT was utilized to determine somatic hypersensitivity by perpendicularly stimulating the center of the posterior paw for less than 4 s with a von Frey filament at 2 weeks after TNBS induction. Raising or biting the stimulated paw was defined as a positive response. The lowest load was 2 g; the highest load, 15 g. Each stimulus was repeated five times, with a 30-s gap between successive measurements.

A thermal stimulator was used to determine TWL, the interval between the beginning of the thermal radiation and the withdrawal behavior, at 2 weeks after TNBS induction. The stimulation was conducted with a preset intensity, which was reduced after 20 s to prevent thermal damage to the skin.

### Western blotting (WB)

Frozen tissues from the spinal dorsal horn were lysed and protein concentration was determined. Proteins were separated by using 10% SDS-PAGE and blotted onto PVDF membranes. Membranes were blocked with 5% milk for 1 h at 25° (± 2°) C and then incubated with primary antibodies at 4 °C overnight. Secondary antibodies against DHAC2 and Actin were used. The membranes were then incubated with horseradish peroxidase-linked secondary antibodies (Amersham) for 1 h at 25° (± 2°) C, and chemiluminescent detection was then performed.

### Extraction of RNA and real-time polymerase chain reaction (PCR)

Trizol reagent (provided by Invitrogen) was used to extract RNA from tissues. LightCycler 480 real-time PCR system was employed to examine mRNA (provided by Roche, Germany), with GAPDH set as the internal control. Quantitative PCRs (Q-PCRs) were performed in 20 μL reaction volumes that contained SYBR Green PCR Master Mix. Transcription levels were detected and the 2^-ΔΔCT^ method was used.

### Construction and transfection of miR-34a inhibitor adenoviral vector

According to a previous method [9, 23], an adenoviral vector was successfully constructed by using Genechem (Shanghai, China). Adenoviruses expressing Scramble (5′-UGU GCA AAU CCA UGC AAA ACU GA-3′, Ad-NC inhibitor-GFP) or the miR-34a inhibitor (5′-UGG CAG UGU CUU AGC UGG UUG U-3′, Ad-miR-34a inhibitor-GFP) were produced Based on the results of a plaque assay, viruses were concentrated to ca. 1 × 10^9^ PFU/mL. For the infection of adenoviruses, a segment of an injured rat common carotid artery was infused with a 100-μL solution containing 1 × 10^9^ PFU/mL Ad-control, 1 × 10^9^ PFU/mL Ad-miR-34a inhibitor, or PBS, and incubated for ca. 0.5 h. Next, the microvascular clips on the internal and common carotid arteries were loosened to ligate the external carotid artery and restore blood flow. The rats were euthanized 2 weeks post treatment and then TNBS was utilized to induce CIVP.

### Intrathecal administration of adenovirus

The PBS buffer (10 μl) was used to resuspend Adenovirus for injection. Mice received an intrathecal injection of 1 × 10^8^ PFU/mL Ad-control and 1 × 10^8^ PFU/mL Ad-miR-34a inhibitor at L5–L6 lumbar vertebrae levels every two days (3 injections) as described previously [24]. At 2 weeks after administration, TNBS was utilized to induce CIVP.

### Dual-luciferase reporter assay (DLRA)

The HDAC2 3′-untranslated region (UTR, − 676~ − 656) was amplified. The binding site of the HDAC2 gene and the miR-34a were removed via site-directed mutation and was used as a control. HEK293T cells were subjected to co-transfection with miR-34a mimic or a negative control mimic using luciferase reporter vectors, and the luciferase assay was performed.

### Statistical analysis

All data were described as mean ± SD. A Student’s t-test or one-way analysis of variance followed by turkey post-hoc test was employed. A *p*-value of < 0.05 (two-tailed) indicated statistical significance.

## Results

### Effects of DEX treatment on AWR, MWT, and TWL of CIVP rats

**Fig. 1:**
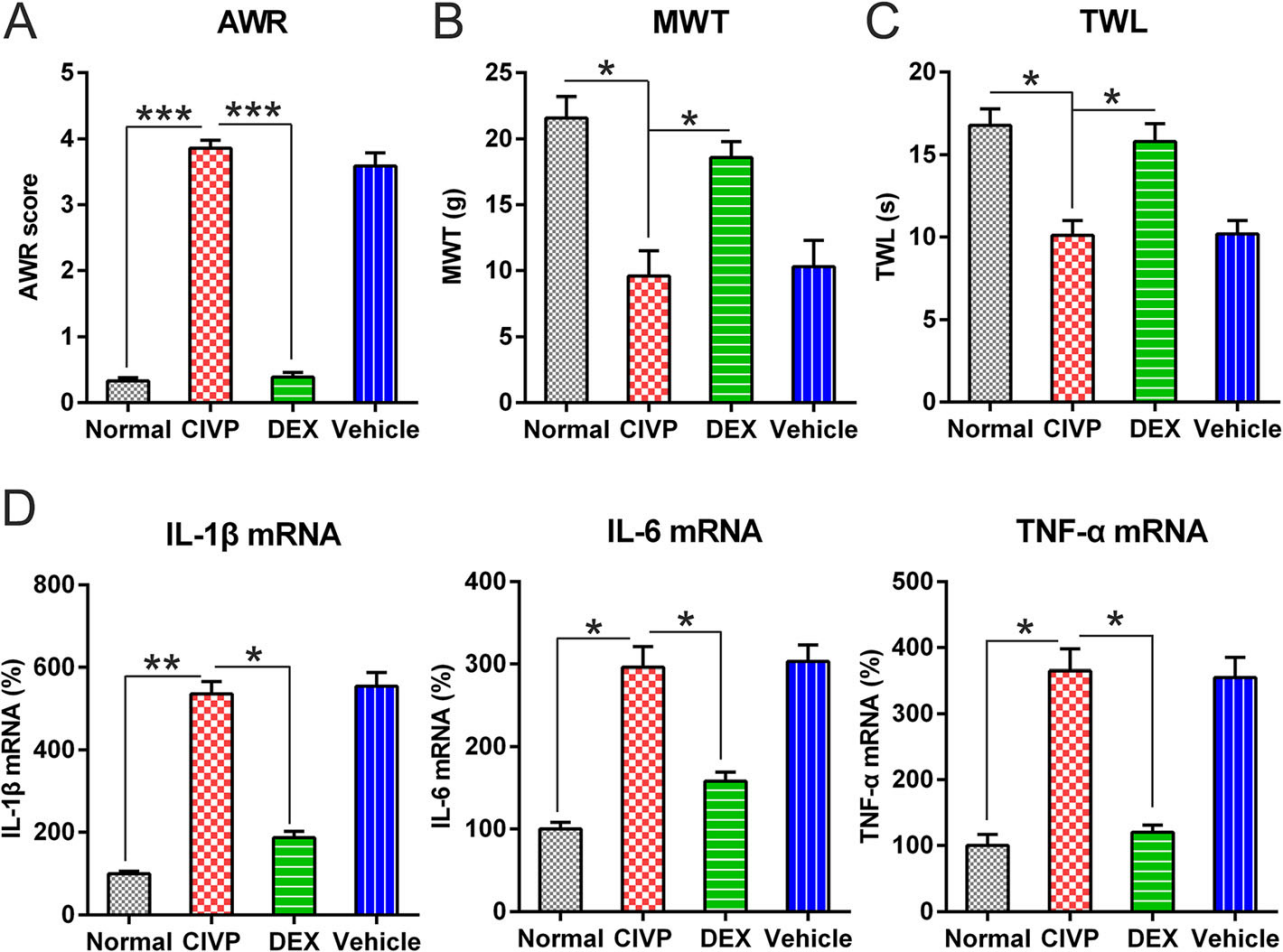
The antinociceptive and anti-inflammatory effects of DEX administration in the TNBS-induced CIVP rat models. **a** AWR figures after DEX treatment for CIVP rat models. **b** MWT following DEX treatment. **c** TWL scores following DEX treatment. **d** Spinal cord was collected from the rats after administration of TNBS. Tissues were then homogenized for IL-1β, IL-6, and TNF-α quantification at mRNA levels via Q-PCR. The results of the three separate tests were recorded as mean ± standard deviation. *N* = 3. *P < 0.05, **P < 0.01, ***P < 0.001, compared to the normal group or the indicated group. Abbreviations: AWL, abdominal withdrawal reflex; CIVP, chronic inflammatory visceral pain; DEX, dexmedetomidine; miRNA, micro ribonucleic acid; MWT, mechanical withdrawal threshold; Q-PCR, quantitative polymerase chain reaction; TNBS, 2,4,6-Trinitrobenzenesulfonic acid; and TWL, thermal withdrawal latency

After initiating CIVP, the AWR scores of treated animals significantly increased relative to those of normal controls (Fig. 1a). By contrast, TWL and MWT scores of treated animals were significantly decreased compared to those of untreated animals (Fig. 1b, c).

DEX administration induced lower AWR scores in the DEX group relative to those in the CIVP and vehicle groups (Fig. 1a). Additionally, MWT and TWL scores were elevated in the DEX group compared to those in the CIVP and vehicle groups (Fig. 1b, c).

### Effects of DEX treatment on inflammatory responses

To evaluate the influence of DEX on the production of pro-inflammatory cytokines, animals were treated with various doses of DEX or vehicle, followed by induction of an inflammatory response by TNBS. Q-PCR was performed to assess the generation of pro-inflammatory cytokines, such as TNF-α, IL-6, and IL-1β, under these conditions. As shown in Fig. 1d, compared to the control groups, expression levels of IL-6, TNF-α, and IL-1β significantly increased in the CIVP group. However, pre-treatment with DEX resulted in an apparent down-regulation of IL-6, TNF-α, and IL-1β expressions. These results demonstrate that inflammation induced by TNBS was ameliorated by prior administration of DEX.

### MiR-34a is downregulated in spinal cord tissues

**Fig. 2:**
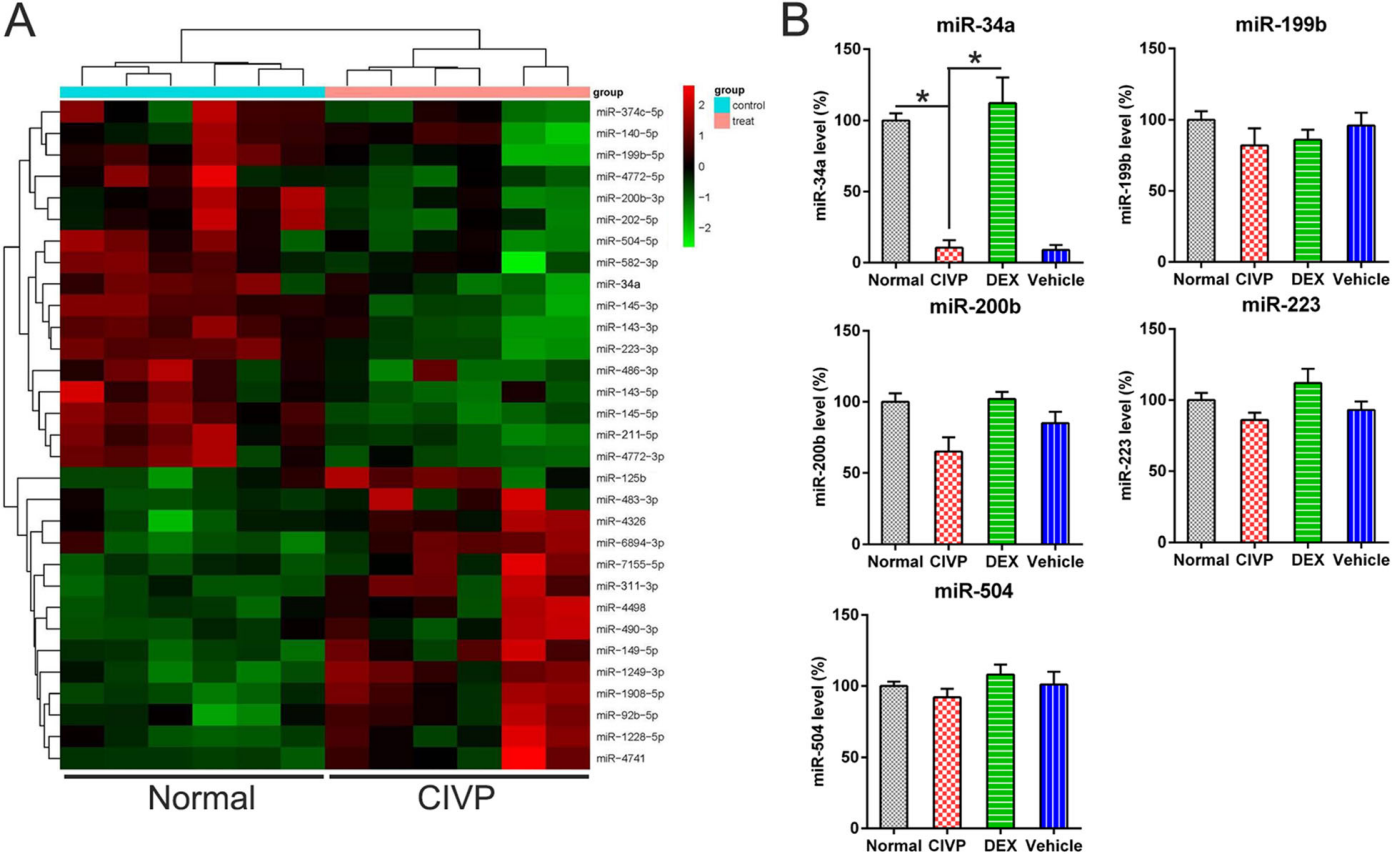
The expression of miR-34a in spinal cord tissues of CIVP rats. **a** miRNA microarray of deregulated miRNAs in spinal cord tissues of CIVP rats treated with DEX or not treated with DEX. **b** Q-PCR detection confirmed the expression of miR-34a, 199b, 200b, 223, and 504 in the normal, CIVP, DEX, and vehicle groups. The data are expressed as mean ± standard deviation (*N* = 3). *P < 0.05, compared to the indicated group. Abbreviations: CIVP, chronic inflammatory visceral pain; DEX, dexmedetomidine; miRNA, micro ribonucleic acid; and Q-PCR, quantitative polymerase chain reaction

The results of the MiRNA microarray revealed a significant downregulation of miR-34a, 199b, 200b, 223, and 504 in the spinal cord tissues of CIVP rats (Fig. 2a). To confirm this data, Q-PCR analysis was performed. Lower miR-34a levels were observed in the spinal cord cells of the CIVP group than in the normal group. Additionally, a significant elevation in miR-34a expression was observed in the DEX group (Fig. 2b) relative to that observed in the CIVP group. By contrast, the levels of other miRNAs were not significantly different among the groups.

### Effect of miR-34a inhibition on pain behaviors of CIVP rats and pro-inflammatory cytokine production

**Fig. 3:**
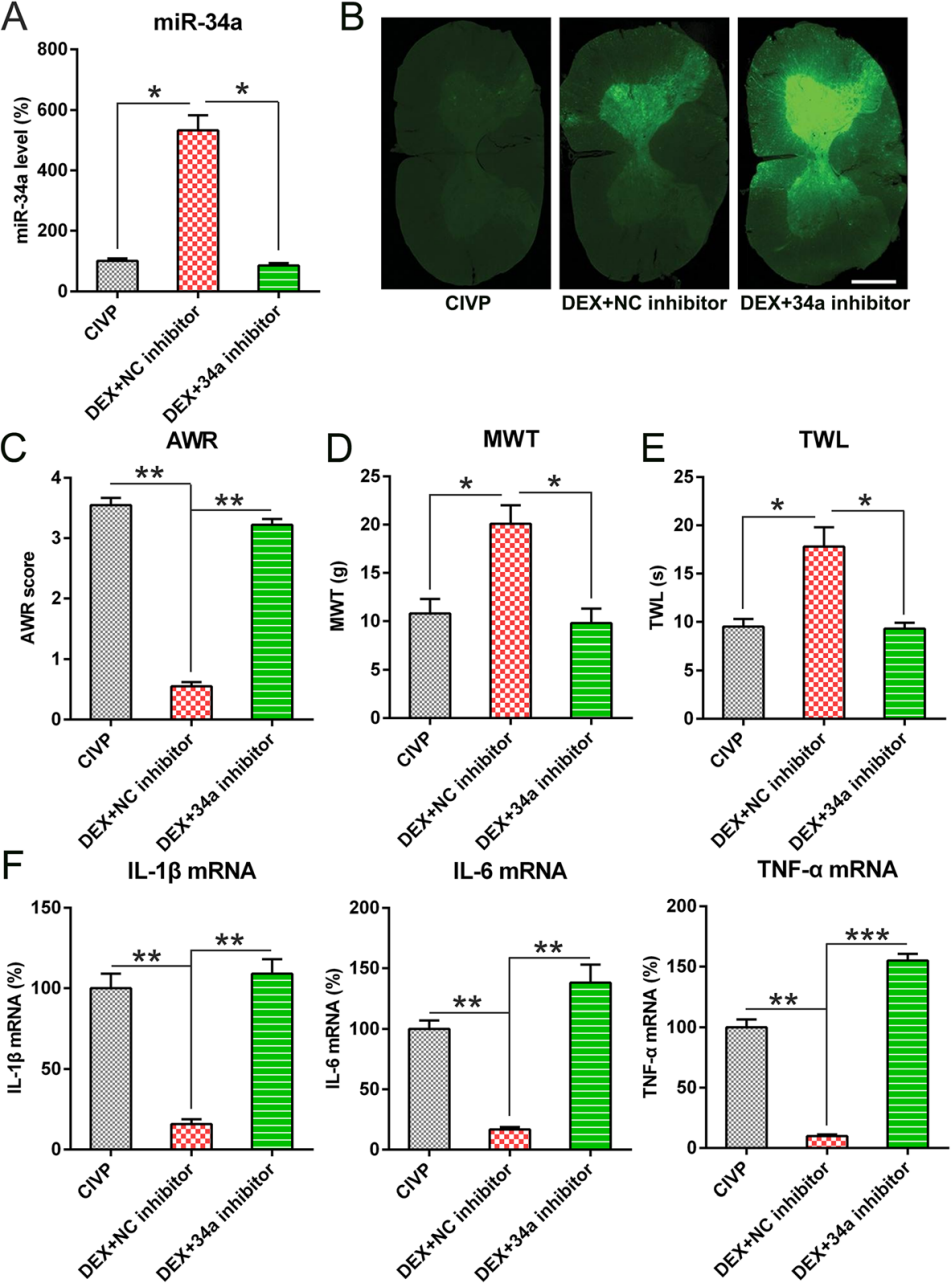
The effect of the inhibited expression of miR-34a on pain behaviors and pro-inflammatory cytokine production of CIVP rats. At 2 weeks after induction with TNBS, spinal cords were isolated from the normal rats and those transfected with adenovirus-miR-34a inhibitor or adenovirus-NC inhibitor. **a** Q-PCR was used to determine the levels of miR-34a in spinal cord tissues. **b** Rats were injected with Ad-NC inhibitor -GFP or Ad-miR-34a inhibitor-GFP with 1 × 10^9^ PFU/mL. The expression of GFP was located and found on the transverse sections of spinal cord. Scale bar: 50 μm. **c**, **d**, **e** MWT, AWR, and TWL scores following DEX treatment and adenovirus infection. **f** Q-PCR was employed to detect mRNA levels of IL-6, IL-1β, and TNF-α and after DEX administration and adenovirus infection. Mean ± standard deviation (*N* = 3) was used to describe the data. *P < 0.05, **P < 0.01, ***P < 0.001, compared to the normal group or the indicated group. Abbreviations: AWL, abdominal withdrawal reflex; CIVP, chronic inflammatory visceral pain; DEX, dexmedetomidine; miRNA, micro ribonucleic acid; MWT, mechanical withdrawal threshold; Q-PCR, quantitative polymerase chain reaction; TNBS, 2,4,6-Trinitrobenzenesulfonic acid; and TWL, thermal withdrawal latency

To probe the influence of miR-34a on DEX-mediated pain behaviors of CIVP rats and pro-inflammatory cytokine production, rats in the DEX groups were infected with adenoviruses containing an miR-34a inhibitor. Q-PCR was employed to determine miR-34a expression. We found that infection with adenovirus-miR-34a inhibitor significantly reduced miR-34a expression in DEX + miR-34a inhibitor groups (Fig. 3a). GFP fluorescence was detected in the spinal cord of rats with injection of adenovirus-miR-34a inhibitor-GFP or adenovirus-NC inhibitor-GFP, while the spinal cord of normal rats did not show GFP fluorescence (Fig. 3b), indicating a high adenoviral transduction.

Rats were also infected with an adenovirus-miR-34a inhibitor. AWR scores in the DEX + miR-34a inhibitor groups were higher than those in the DEX groups (Fig. 3c). The DEX + miR-34a inhibitor also effected lower TWL and MWT than did DEX administration alone (Fig. 3d, e), indicating that miR-34a inhibits pain behaviors in CIVP rats.

The production of pro-inflammatory cytokines was measured to study the role of miR-34a inhibition in CIVP rats that received DEX. The mRNA levels of these three cytokines were significantly upregulated in the DEX + miR-34a inhibitor group relative to the DEX group (Fig. 3e), suggesting that miR-34a plays a role in the DEX-mediated TNBS-induced inflammatory responses and pain behaviors.

**Fig. 4:**
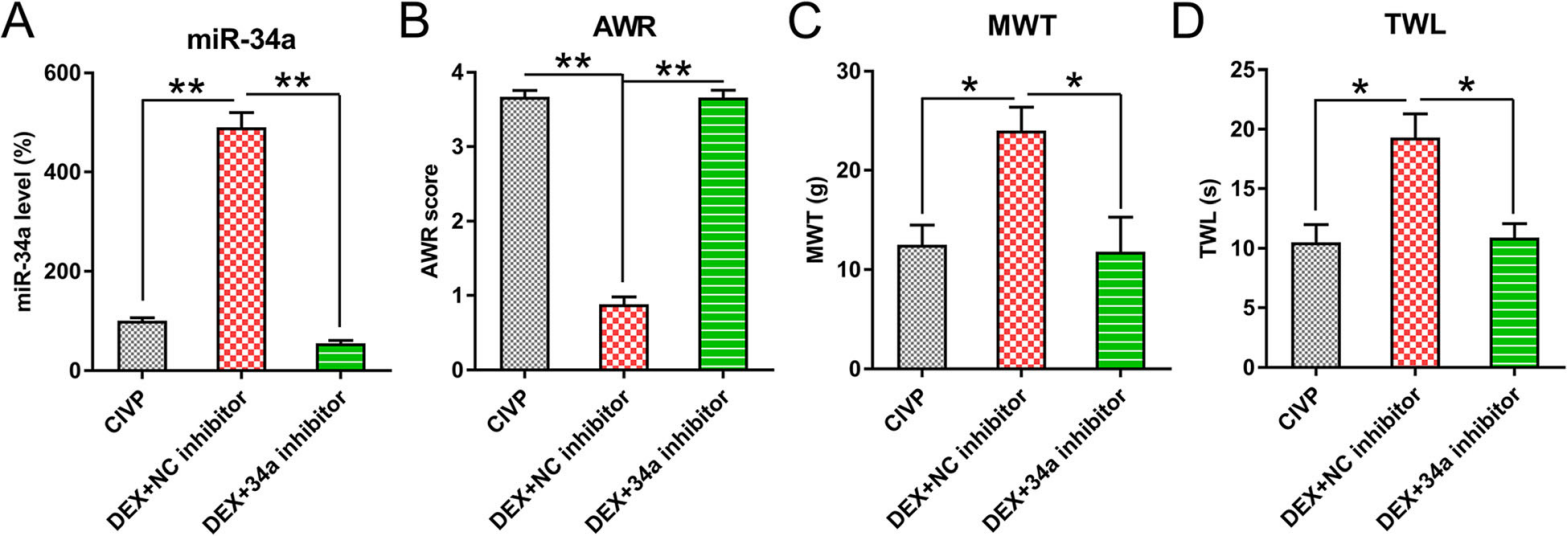
The effect of intrathecal injection of adenovirus-miR-34a inhibitor on pain behaviors of CIVP rats. At 2 weeks after induction with TNBS, spinal cords were isolated from rats with intrathecal injection of adenovirus-miR-34a inhibitor or adenovirus-NC inhibitor. **a** Q-PCR was used to determine the levels of miR-34a in spinal cord tissues. **b**, **c**, **d** MWT, AWR, and TWL scores following DEX treatment and intrathecal injection. Mean ± standard deviation (*N* = 3) was used to describe the data. *P < 0.05, **P < 0.01, ***P < 0.001, compared to the normal group or the indicated group. Abbreviations: AWL, abdominal withdrawal reflex; CIVP, chronic inflammatory visceral pain; DEX, dexmedetomidine; miRNA, micro ribonucleic acid; MWT, mechanical withdrawal threshold; Q-PCR, quantitative polymerase chain reaction; TNBS, 2,4,6-Trinitrobenzenesulfonic acid; and TWL, thermal withdrawal latency

To ascribe the effects of adenovirus to the spinal cord, we further performed intrathecal adenoviral administration to knockdown miR-34a. Q-PCR clearly showed that miR-34a expression was inhibited after adenoviral administration (Fig. 4a). AWR scores in the intrathecal DEX + miR-34a inhibitor groups were increased, comparing with intrathecal DEX + NC inhibitor groups (Fig. 4b). The intrathecal DEX + miR-34a inhibitor also led to reduced TWL and MWT than DEX + NC inhibitor group (Fig. 4c, d), indicating that intrathecal injection of Adenovirus-miR-34a inhibitor repressed pain behaviors in CIVP rats.

### MiR-34a targets 3’UTR of HDAC2

**Fig. 5:**
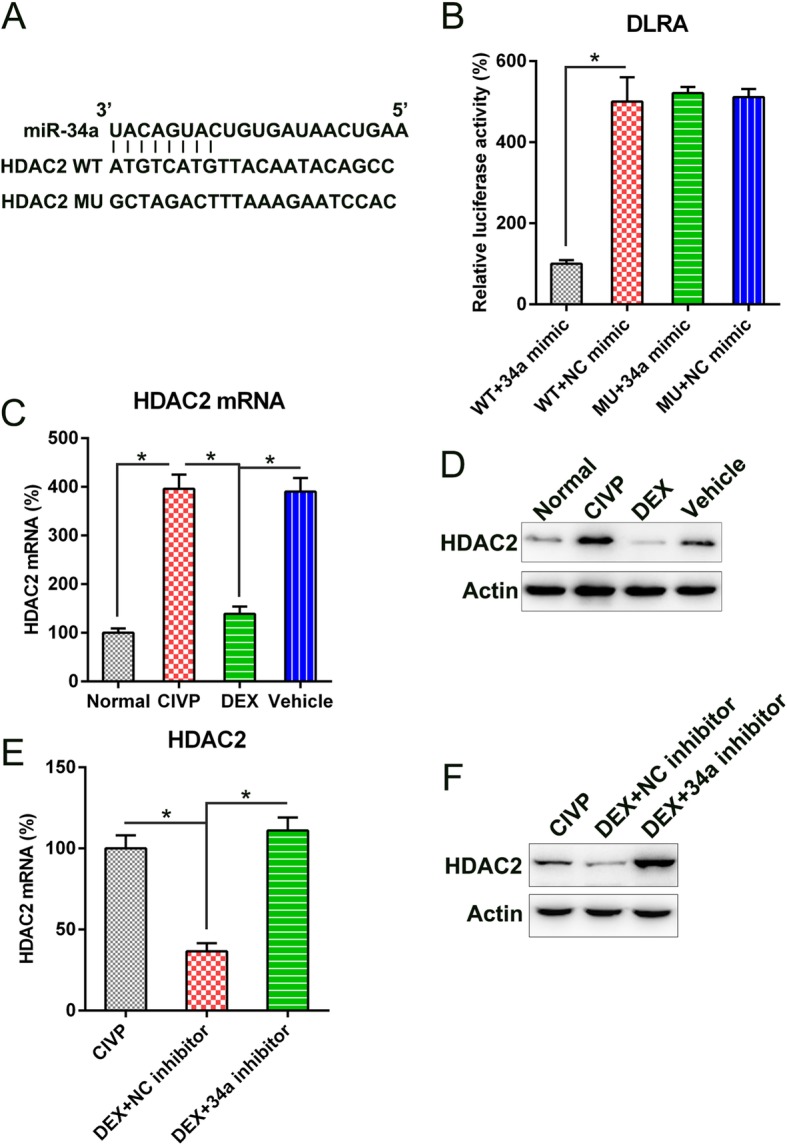
miR-34a targeted the 3′-UTR of HDAC2. **a** Bioinformatics analysis showed that miR-34a has a binding site at the 3′-UTR of the HDAC2 mRNA. **b** DLRA was performed following co-transfection of a luciferase reporter that contained either a mutant (MU) or wild-type (WT) 3′-UTR from HDAC2 and the miR-34a mimic into HEK293T cells. The influence of the miR-34a on the luciferase activities of the MU and WT HDAC2 reporter constructs were examined. **c**, **d** WB and Q-PCR analyses showed the expression of HDAC2 expression in normal, CIVP, DEX, and vehicle groups at both mRNA and protein levels. **e**, **f** WB and Q-PCR analyses showed the expression of HDAC2 expression in CIVP and DEX with or without miR-34a inhibition at both mRNA and protein levels. Results are expressed as mean ± SD. *P < 0.05 as compared to the indicated groups. Abbreviations: CIVP, chronic inflammatory visceral pain; DLRA, dual luciferase reporter assay; DEX, dexmedetomidine; miRNA, micro ribonucleic acid; Q-PCR, quantitative polymerase chain reaction; TNBS, 2,4,6-Trinitrobenzenesulfonic acid; and WB, Western blot

The bioinformatics analysis showed that miR-34a could target the 3′-UTR of HDAC2 (Fig. 5a). We therefore investigated the direct interaction between HDAC2 and miR-34a using DLRA. The direct interaction between miR-34a and the 3′-UTR of HDAC2 was investigated using DLRA (Fig. 5b). The data showed that the luciferase activity was suppressed after the transfection of miR-34a mimic, which was fused with a 3′-UTR of HDAC2, by 70% compared to the other control groups.. The HDAC2 signal reportedly has a significant effect on the modulation of pain effects [25, 26]. We then tested for differences in the expressions of HDAC2 mRNA and protein among the different groups: HDAC2 levels were higher in the CIVP group than in the normal group, and DEX administration decreased the level of HDAC2 at both mRNA and protein levels (Fig. 5c, d). Additionally, the transfection of rats with a miR-34a inhibitor further promoted the expression of HDAC2 in DEX-treated rats relative to thos transfected with an negative control inhibitor (Fig. 5e, f). Our results thus indicate that miR-34a potentially targets the 3′-UTR of DHAC2.

### HDAC2 knockdown (KD) abated the effect of DEX on CIVP rats

**Fig. 6:**
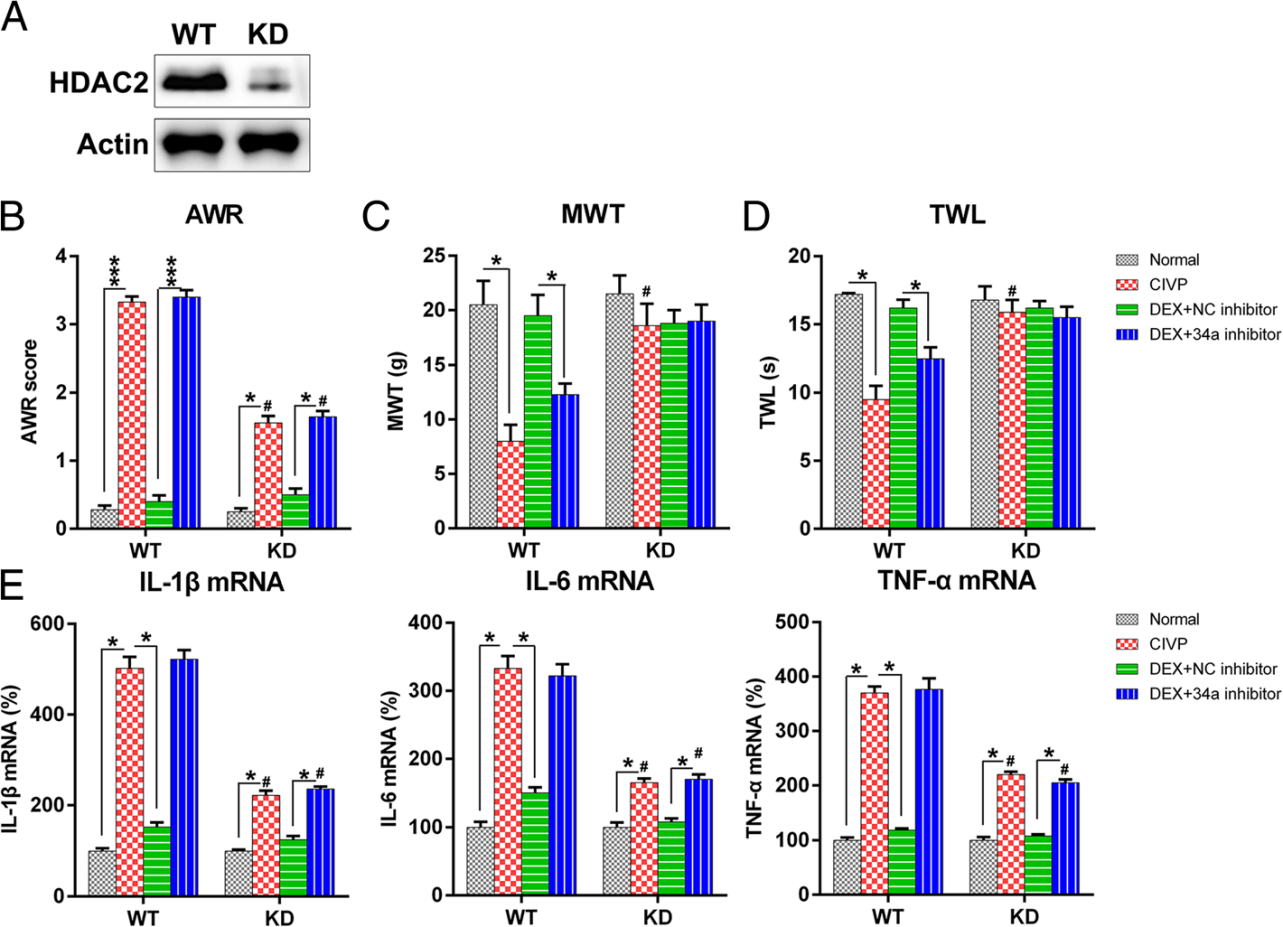
Influence of HDAC2 KD on pain behavior and inflammatory cytokine production of CIVP rats. **a** HDAC2 expressions were examined in spinal cord tissues of WT and HDAC2 KD rats with CIVP. **b** The post-treatment AWR figures. **c** MWT following DEX treatment. **d** TWL scores following DEX treatment of CIVP with different treatment. At 30 min after acetic acid was injected, we collected peripheral blood from the animals, and (**E**) the serum mRNA levels of IL-6, TNF-α, and IL-1β were determined by Q-PCR after DEX administration and adenovirus infection. Mean ± standard deviation (*N* = 3) was used to describe the data. *P < 0.05, ***P < 0.001, compared to the indicated group. ^#^*P* < 0.05, compared to same treated group of WT rats. Abbreviations: AWL, abdominal withdrawal reflex; CIVP, chronic inflammatory visceral pain; DEX, dexmedetomidine; KD, knock down; miRNA, micro ribonucleic acid; MWT, mechanical withdrawal threshold; Q-PCR, quantitative polymerase chain reaction; TWL, thermal withdrawal latency; and WT, wild type

To further confirm the importance of HDAC2 in DEX-treated CIVP rats, we used a HDAC2 KD rat to create the CIVP model. The CIVP HDAC2 KD rat underwent the same DEX treatment, and a wild-type (WT) rat was used as the control. WB was performed to confirm the reduction of HDAC2 in KD rats (Fig. 6a). Pain behavior tests were performed, and the spinal cord of the rats were isolated and analyzed with Q-PCR to examine the pro-inflammatory cytokine production. To elucidate the effect of HDAC2 on DEX-mediated pain behavior in the CIVP rats, the same behavior tests were also performed. We found lower AWR scores in the CIVP and DEX + miR-34a inhibitor groups with HDAC2 KD rats than in those with WT rats; however, DEX treatment still resulted in the significant alteration of AWR scores in the latter (Fig. 6b). Notably, TWL and MWT values also increased significantly in the CIVP KD rats relative to the CIVP WT rats. However, DEX treatment did not significantly alter TWL and MWT values in KD rats (Fig. 6c and d). These data suggest that DEX administration influences the pain behaviors of CIVP rats, at least in part, through HDAC2 signaling.

To further determine the influence of HDAC2 expression on the inflammation of CIVP rats, we examined the generation of pro-inflammatory cytokines within each group of HDAC2 KD rats. Q-PCR was performed to assess IL-1β, TNF-α, and IL-6 at the protein level. As shown in Fig. 6e, expression levels of the three cytokines were significantly reduced for both WT and HDAC2 KD rats after DEX treatment. Notably, however, the extent of reduction in HDAC2 KD rats was significantly less than that in WT rats. Meanwhile, pre-treatment with DEX also resulted in an apparent downregulation of IL-1β, IL-6, and TNF-α in the HDAC2 KD rat relative to the WT rats. These results demonstrate that the acute inflammation downregulated by DEX was partially dependent on HDAC2 expression.

## Discussion

Frequent CIVP is common among patients who suffer inflammatory bowel disease; however, estimating CIVP levels is challenging on account of a dearth of knowledge regarding the localization, expansion, and pathogenesis of CIVP. Evaluating the analgesic efficacy, abdominal contractions, or AWR comprise the current means of evaluating CIVP [27], despite their being increasingly regarded as inadequate diagnostic assays. Our study showed that TNBS stimulation of the bowels produces CIVP. However, visceromotor behavioral responses to pain were diminished by DEX administration. While TNBS stimulation was also found to decrease miR-34a levels in the spinal cord, DEX administration restored the expression of miR-34a. In addition, we showed that TNBS stimulation increased HDAC2 expression, which was subsequently attenuated by DEX administration. DLRA data demonstrated the ability of miR-34a to target the 3′-UTR of the HDAC2 gene. In CIVP rat models, the inhibited expression of miR-34a recovered the effect of DEX on TNBS-induced CIVP, as evinced by pain behavior tests and the concentrations of pro-inflammatory cytokines. In addition, we found that CIVP was more moderate in HDAC2 KD rats than in WT rats. Although previous models have demonstrated the analgesic potential of DEX [28, 29], our study provides novel evidence for the influence of DEX on CIVP, as well as for possible underlying mechanisms this effect.

HDAC2 has been widely reported to participate in the regulation of multiple biological reactions in various malignancies [30–34]. However, the possible impact of HDAC2 on visceral pain is unclear. Chronic pain is a complicated clinical manifestation that undermines the quality of life for billions of people. The effects of epigenetic modulation, i.e., the modification of histone proteins and/or DNA, on neuronal plasticity has garnered increasing attention pain research. Research on epigenetic modulation has shown that algesia-related synaptic plasticity is regulated by changes in histone acetylation levels and HDAC activity. Hence, HDACs play an important role in the development and persistence of chronic pain [35]. Denk et al. found that the intrathecal delivery of various class I HDACIs and antiretroviral drugs (stavudine, d4T) into the spinal cords of rats with traumatic nerve injury induced peripheral neuropathy. Thermal and mechanical hypersensitivity was decreased by 40–50% after HDACI treatment, but only if administered prior to any insult. Histone acetylation was promoted by drug delivery to the spinal cord, but it exerted no obvious effect on the related dorsal root ganglia [36]. It was reported that the HDAC inhibitor trichostatin A evinced antinociceptive activity on bone cancer pain by restoring μ-opioid receptor expression [36]; expressions of certain HDACs were ascertained, revealing that the HDAC2 expression was enhanced in the spinal cord tissues in a time-dependent manner and that trichostatin inhibited HDAC2 expression in PC12 cells. HDAC2 silencing further indicated that HDAC2 was significant for mechanical hyperalgesia modulation after the inoculation of tumor cells. Notably, μ-opioid receptor expression was not restored by HDAC2 KD, but the decreased potassium-chloride cotransporter 2 was reversed. Reduced potassium-chloride cotransporter 2 expression is thought to be an important mechanism causative of pathological pain. HDAC2 in the spinal cord contributed to mechanical hyperalgesia in a murine model of bone cancer pain. This report proves that HDAC2 expression is inhibited by miR-34a; the increased HDAC2 expression following miR-34a inhibitor transfection impaired the effect of DEX on CIVP, and HDAC2 KD attenuated CIVP. These results are consistent with those of previous studies. Furthermore, we demonstrated an increase in the stimulation of the JAK/STAT axis in LSCs that were impaired by the expression of p53 and miR-34a.

## Conclusion

Our data show that DEX administration inhibits TNBS-induced inflammatory visceral pain and has a significant inhibitory effect on the production of CIVP-associated pro-inflammatory cytokines in murine CIVP models. Injection of DEX increases the level of the miR-34a and decreases the expression of HDAC2. Our study also suggests that the antinociceptive impact of DEX may be regulated through the suppression of the inflammatory response through miR-34a-mediated involvement of HDAC2 in CIVP.

## Data Availability

The datasets used and/or analyzed during the current study are available from the corresponding author on reasonable request.

## Abbreviations

AWR: Abdominal withdrawal reflex
CIVP: Chronic inflammatory visceral pain
CRD: Colorectal distending
DEX: Dexmedetomidine
DLRA: Dual-luciferase reporter assay
KD: Knockdown
miRNAs: Micro ribonucleic acids
MWT: Mechanical withdrawal threshold
PCR: Polymerase chain reaction
Q-PCRs: Quantitative PCRs
SD: Sprague Dawley
TNBS: Trinitrobenzenesulfonic
TWL: Thermal withdrawal latency
UTR: Untranslated region
WB: Western blotting
WT: Wild-type
